# Elements of the niche for adult stem cell expansion

**DOI:** 10.1177/2041731417725464

**Published:** 2017-08-24

**Authors:** Patricia A Redondo, Marina Pavlou, Marilena Loizidou, Umber Cheema

**Affiliations:** 1 Division of Surgery and Interventional Science, University College London, London, UK; 2 Institute of Orthopaedics & Musculoskeletal Science, University College London, London, UK

**Keywords:** Biomimicry, adult stem cells, microenvironment

## Abstract

Adult stem cells are crucial for tissue homeostasis. These cells reside within exclusive locations in tissues, termed niches, which protect adult stem cell fidelity and regulate their many functions through biophysical-, biochemical- and cellular-mediated mechanisms. There is a growing understanding of how these mechanisms and their components contribute towards maintaining stem cell quiescence, self-renewal, expansion and differentiation patterns. In vitro expansion of adult stem cells is a powerful tool for understanding stem cell biology, and for tissue engineering and regenerative medicine applications. However, it is technically challenging, since adult stem cell removal from their native microenvironment has negative repercussions on their sustainability. In this review, we overview specific elements of the biomimetic niche and how recreating such elements can help in vitro propagation of adult stem cells.

## Introduction

Advances in the knowledge of stem cell biology have resulted in new approaches to treat human disease. This holds enormous potential to revolutionize medicine through solutions for conditions that require tissue regeneration. However, much remains to be understood in terms of stem cell behaviour and technical optimization of protocols to allow proper translation into the clinical setting. The two main fronts of research and clinical application of stem cells comprise (1) stem cell therapies, most commonly based on cell injections or tissue engineered grafts, in which scaffolds are used in combination with stem cells and biologically active molecules and (2) in vitro studies for basic biology research and drug testing.

The application of cell-based strategies is highly limited by difficulties in harvesting and expanding stem cells. For this reason, the most extensively studied cells are embryonic stem cells (ESCs) and induced pluripotent stem cells (iPSCs) that have the ability to differentiate into all adult tissues and to thoroughly proliferate, as well as multipotent mesenchymal stem cells (MSCs), obtained in clinically relevant amounts with potential to differentiate into some mesodermal-derived cell types. Nevertheless, the former present disadvantages such as the risk of teratoma formation and ethical controversies, while the latter cannot be applied to many tissues in the body nor be studied as tissue-specific stem cells in vitro.^1,2^ Tissue-specific adult stem cells (ASCs), however, give rise to all cell types from their given lineage and have been identified in most tissues of the human body.^3^ The native three-dimensional (3D) environment that surrounds ASCs is composed of extracellular matrix, neighbouring cells and soluble factors. Within these dynamic microenvironments, denominated niches, ASCs are subjected to biochemical, biophysical and biomechanical phenomena that sustain their ability to both self-renew and differentiate, also known as stemness.^4^ Functionally, ASCs would be ideal candidates for regenerating injured tissues, since they are natively responsible for maintaining tissue homeostasis throughout life.^5^ Yet, their isolation and expansion must be substantially improved to allow a better understanding of their identity and behaviour in vitro, as well as to achieve a more effective manipulation for translational use in the clinical setting. Aiming to overcome the challenges of expanding ASCs taken away from their native microenvironment, strategies have been developed to mimic the niches in which these cells reside.

In this review, we will discuss the different features of the biomimetic niche that promote tissue-specific ASC expansion, using haematopoietic, brain, intestinal and muscle tissues as examples. Since the topics of MSC, ESC and iPSC expansion, as well as biomimetic systems promoting differentiation, have been widely covered in the literature, we will not approach such themes, but will review tangible qualities of the biomimetic niche that are necessary for ASC expansion in vitro.

## ASCs and their microenvironment

Stem cells reside in vivo within tissue-specific niches where multiple interactions occur. They have the ability to self-renew for long periods and to differentiate into more committed lineages respective of their host tissue.^3^ Together, cell-to-cell and cell-to-matrix interactions, soluble molecules signalling, physical and mechanical stimuli determine if a stem cell will remain quiescent, divide symmetrically (self-renew) or asymmetrically, producing one cell with same potency and one more differentiated cell.^6^

When considering the need for stem cells expansion, it is necessary to take into account the native behaviour of the known stem cell populations within a tissue. Tissues in the body have different turnover rates and regenerative potential. For instance, gut, mammary, blood and skin tissues have both a high turnover and regenerative capacity, completely replacing their differentiated cell population within a matter of days or weeks. However, tissues like liver and skeletal muscle present low steady-state turnover rates, but highly effective repair abilities, swiftly recruiting ASCs or progenitor cells in response to injury or disease in order to repair and regenerate. Other tissues such as the brain and heart have low turnover rates and poor repair upon damage, with resident stem cells dividing rarely.^7,8^

The ASC niches change when ASCs face challenging physiological events, such as mammary gland alterations during pregnancy and hair follicle variations during hair cycle, which require massive cell proliferation, apoptosis and differentiation. Different cell populations are required to adjust to these changes, including more dormant stem cells, usually recruited only during critical need, as well as stem cells with a higher cell cycle rate, more generally associated with tissue homeostasis.^9^

The existence of a stem cell niche that regulates self-renewal and differentiation was postulated for the human haematopoietic system three decades ago.^10^ Current knowledge hypothesizes that there are two haematopoietic stem cell (HSC) niches in the adult bone marrow. The osteoblastic niche on the bone surface maintains a quiescent environment for long-term HSCs (LT-HSCs) via endosteal osteoblasts. When mobilized, during haematopoiesis, LT-HSCs give rise to short-term HSCs (ST-HSCs), which are more proliferative and able to generate differentiated blood progenitors. This cell behaviour is promoted by their location, adjacent to sinusoid endothelial cells, within a vascular niche.^11,12^

The subgranular zone (SGZ) in the dentate gyrus (DG) of the hippocampus and the subventricular zone (SVZ) of the lateral ventricles are the main neurogenic regions in the adult brain. In the SVZ, quiescent neural stem cells (NSCs), known as B-type cells, become activated, generating transit amplifying cells (C-type) which will give rise to type-A neuroblasts or glial cells.^13^ In the SGZ of the DG, new granule neurons and astrocytes are continuously generated by neural progenitor cells (NPCs).^14^

The intestinal epithelium is sustained by leucine-rich repeat containing G-protein coupled receptor 5 (Lgr5+) cells that reside at the bottom of the crypts. In the small intestine (SI), they are interspersed by Paneth cells, which function as their niche, providing intestinal stem cells (ISCs) with necessary signals.^15^ In the colon, the stem cells are intermingled with Reg4+ deep secretory cells (DSCs) that act similarly to Paneth cells.^16^

Satellite cells (SaCs) are quiescent cells in the muscle, located below the basal lamina of the muscle fibre. Generally considered the muscle stem cells (MuSCs), they are capable of self-renewal, maintaining the SaCs pool and entering the cell cycle when required by external stimuli such as injury and exercise, producing myogenic precursor cells.^17^

Growing ASCs has been extremely challenging due to the elaborate spatio-temporal arrangement of multiple factors that regulate activation, sustained proliferation and maintenance of an undifferentiated state in vitro. Recreating the critical features to either maintain multipotency or cause controlled specific differentiation is a strategy employed by many researchers in the field.

Two-dimensional (2D) culture is often simplistic and lacks the necessary complexity for stem cell growth, as seen by tentative unsuccessful expansion of cells in these systems.^18^ 3D structures mimicking biological, mechanical and physical properties of ECM are promising systems for advancing such cultures.^19,20^ Recently, more complex biomaterials for improving ASC proliferation and control of stem-cell fate have been developed, as well as an appreciation of the soluble factors that control these processes.

The importance of niche composition for ASC expansion can be illustrated by parabiotic studies that show SaCs and liver progenitors from aged mice restoring their regeneration capacities when exposed to blood-borne niche factors from younger mice. This indicates that the intrinsic replicative ability of these progenitors may not be the sole cause of tissue degeneration during ageing.^21,22^ Similar results were observed with NPCs expansion and related cognitive function.^23^ Furthermore, the niche size regulates the number of ASCs in the bone marrow. An increase in the number of osteoblastic cells to which LT-HSC attach promotes their expansion,^24^ while niche cell depletion causes LT-HSC diminishment.^25^

Although stem cell niches vary among tissues, they share common microenvironmental features. Delineating these features and the mechanisms by which cell fate is controlled through biophysical and biochemical parameters within the niche may enhance current ASC expansion strategies.

## Microenvironment components of ASC niches

The molecular signalling pathways involved in ASC regulation and self-renewal have been studied extensively. The ASC niche is a dynamic system where the equilibrium between stem cells and their microenvironment is mutually maintained via multiple interrelated communication networks. This multidirectional crosstalk involves not only the biochemical cues exchanged between ASCs, their surrounding cells and ECM, but also a physical relationship. There is a growing appreciation of the biomechanical and biophysical roles in the maintenance of the stem cell niche. Niche homeostasis relies heavily on biophysical properties, such as ECM physicochemistry, pH, fluid dynamics, oxygen tension and mechanical properties of the originating tissue.^26,27^

### Biochemical factors

In the stem cell niche, there is a plethora of morphogens, chemokines, cytokines and growth factors, present either as soluble molecules or as ECM-bound. The communication among ASC and other cells from the niche is mediated by such biomolecules acting via paracrine, autocrine or juxtacrine signalling pathways. Not only the composition of these chemotactic gradients, but also their spatio-temporal distribution, play a significant role in controlling ASC fate.

#### Wnt

The Wnt pathway is a highly conserved signalling network with crucial functions as a morphogen during development and as a regulator of multiple cellular behaviours throughout life. In mammals, it comprises a family of 19 ligands that interact mainly with the multi-pass membrane receptor Frizzled (diagramatically presented and reviewed in Nusse^28^). In the canonical pathway, it usually requires the involvement of the LPR5/6 co-receptor, leading to the activation of multiple downstream intracellular molecules, exerting its effects by the nuclear accumulation of β-catenin, which in turn promotes transcription of target genes. The planar cell polarity pathway, which affects cell movement, and the calcium pathway are two non-canonical pathways also activated by Wnt ligands.^29^ Wnt signalling has been directly implicated in self-renewal, proliferation and differentiation of ASCs in a tissue-specific manner.

For instance, overexpression of β-catenin has been reported to expand HSCs in long-term cultures and in vivo, showing the Wnt pathway involvement in HSC self-renewal and proliferation via the specific ligand Wnt3a.^30–32^ Furthermore, HSCs have demonstrated high sensitivity to Wnt dosage, indicating that adjustments for in vitro expansion and clinical applications are essential.^33^

Wnt signalling is considered a central regulator of adult neurogenesis in the hippocampus^34,35^ and the SVZ.^35,36^ In a mouse model of β-catenin constitutive activation, Wnt was associated with the expansion of NPCs, resulting in a bigger cortex.^37^ However, Wnt3a conditioned media caused inhibition of neurosphere formation concomitant with neuronal differentiation in cultured NSCs derived from E11.5 mouse forebrain,^38^ whereas it promoted proliferation, but not differentiation, of hippocampal NPCs from E15 mice in another study.^39^ This suggests that Wnt types and protocols should be fine-tuned according to the age and anatomical source of NSCs in order to promote the desired effects in vitro.

Crypt cells in the SI and colon depend on Wnt signalling to proliferate.^40,41^ R-spondin1 (Rspo1), a Wnt agonist that acts via Lgr4/5 receptors, is an important mitogenic factor for crypt cells.^42,43^ Hence, Wnt3a and Rspo1 addition to systems aiming to expand ISCs is crucial.^44–46^

MuSCs also proliferate upon Wnt signalling during regeneration via the canonical pathway, stimulated by Wnt1, Wnt3a, Wnt5a or Wnt11, as opposed to Wnt4 or Wnt6, which inhibits MuSCs proliferation.^47^ Activation of the planar cell polarity pathway by Wnt7a was also reported as myogenic.^48^ Conversely, increased Wnt/β-catenin signalling was found to mediate muscle fibrosis acquired with ageing by promotion of fibrogenic conversion in aged myogenic progenitors.^22^ Taken together, these studies illustrate the importance of precisely tailoring Wnt manipulation to the chosen source of ASCs.

Besides sustaining Wnt signalling via the use of pathway agonists, attention should also be given to inhibitory molecules, such as Dickkopf (Dkk)-related proteins that bind to LRP5/6, preventing the activation of downstream cascades promoted by Wnts.^49^ Studies using these potent inhibitors of Wnt signalling have helped to build further evidence on the key importance of Wnt for ASCs, via disruption either of stemness or of the ASC proliferative compartment in HSCs,^31^ NSCs^50^ and ISCs.^41,51^ Although Wnt addition to culture seems sufficient for maintaining pathway activation in vitro, the use of DKK inhibitors, like NCI8642,^52^ especially in complex models utilizing multiple cell types, remains to be tested for a putative enhancement of ASCs expansion. Such experimental strategies could also be applied to the signalling pathways described in the next sections.

#### Notch

Notch signalling is an evolutionary conserved pathway based on membrane-bound Notch receptors that bind to transmembrane ligands such as Delta (Dll) and Jagged present on adjacent cells, activating transcription of downstream genes via intracellular cascades (comprehensive schematics can be found in in-depth reviews;^53,54^). There is evidence that Notch signalling is involved in stemness maintenance within the four tissue types discussed in this review.

Notch pathway activation has been reported to promote adult HSCs self-renewal in vitro^55^ and in vivo,^56^ regulating both ST-HSCs and LT-HSCs.^57^ However, its role remains to be further elucidated, as Notch signalling has also been described as dispensable.^58,59^

High Notch activity has been associated with maintenance of NSCs quiescence,^60^ NSCs survival and an increase in NPCs accompanying improved functional response to injury. Therefore, Notch is considered to play an essential role in neurogenesis.^61,62^

The Notch pathway is crucial for preventing ISCs from differentiating.^63^ Hence, Dll4 signalling is indispensable for ISC culture,^45^ consonant to in vivo observations, where Notch ablation in mice led proliferative crypt cells to differentiate into post-mitotic goblet cells.^64^

The quiescent MuSCs pool was diminished when Notch signalling was genetically ablated.^65,66^ Correspondingly, upregulation of Notch stimulated MuSCs proliferation in response to injury.^67^

With the exception of HSCs, for which Notch signalling literature reports are conflicting, evidence suggests that this pathway activation generally promotes ASC proliferation.

#### BMP

Bone morphogenetic proteins (BMPs) belong to the TGF-β superfamily of ligands that interact canonically with type I and type II receptors which, in turn, transduce their signals through the phosphorylation of Smad, resulting ultimately in gene expression regulation (reviewed in Wang et al.^68^). In ASCs, BMP signalling has been widely implicated in the control of self-renewal.

Ex vivo HSC culture time, for example, was extended by the addition of BMP4 to the media.^69^ However, the use of a deletion model of the BMP1Ra receptor revealed that BMP signalling disruption led to an increase in LT-HSCs in mice via the expansion of the osteoblastic cells to which they attach.^24^

In hippocampal NSCs, BMPR1a activation via BMP induced quiescence.^70^ The addition of the BMP antagonist noggin to in vitro cultures of hippocampal NSCs augmented expressively their cell number and maintenance, while in SVZ NSCs, it only accelerated expansion, without an increase in the cell population.^70^ Although expansion can be achieved by abrogating BMP signalling with noggin, this may lead to exhaustion of the stem cell pool.^71^ Nevertheless, inhibition of NPC proliferation mediated by age-related enhanced BMP signalling could be partially restored in mice by the blockade of Smad. Blockade of excessive BMP signalling found during ageing may be, then, beneficial to health, as long as it is not strong enough to extinguish the NSC pool as seen in younger adults.^72^

Correspondingly, self-renewal of ISCs was suppressed by BMP crosstalk and blockade of the Wnt pathway.^73^ BMP activity towards intestine cell differentiation has been shown to be abrogated by noggin,^74,75^ promoting ISCs expansion in culture when added to the media.^44,45^

Within the TGF-β superfamily pathways, the most studied in MuSCs is the inhibitory effects of TGF-β1 on proliferation, impairing muscle regeneration via Smad3.^76–78^ However, BMP signalling in vivo and in vitro was shown to stimulate proliferation,^79^ preventing cell cycle exit of activated SaCs and proliferating myoblasts, resulting in MuSCs expansion.^80^ Thus, it is critical to consider not only the pathways in which to interfere with when optimizing ASC expansion in vitro but also the multiple molecular targets which may lead to the desired outcomes.

Other morphogens like Sonic hedgehog (Shh) have also been shown to be involved in ASCs regulation. Its actions have been studied in more depth within the brain tissue, where Shh was required for NSC maintenance.^61,81,82^ Nevertheless, its function remains to be clarified, as Shh has also been shown to promote NSCs specification to neurons.^83^ In the intestine, crypts of Shh null mice were hyperplastic,^84^ exhibiting increased proliferation,^85^ suggesting a role for Shh in ISC proliferation.

It is important to note, though, that all these events take place within cells and their niches in a dynamic fashion. Several studies suggest the existence of communication and crosstalk amongst these major signalling pathways,^61,73,86^ which may account for the controversies found when studying these molecules separately, as well as during in vitro modelling.

#### Growth factors, chemokines, cytokines and other molecules

Growth factors within tissues are either bound to the ECM or secreted by stem cells and other niche cells. The regulation of growth factor availability is tightly controlled in vivo. While morphogens tend to sustain stem cells at an undifferentiated state, concomitant action of growth factors generally triggers proliferation via signal transduction cascades using tyrosine kinase pathways.^28^

In vitro culture and expansion of HSCs is controlled by addition of specific cytokine cocktails, which include stem cell factor (SCF), FMS-like tyrosine kinase-3, thrombopoietin (TPO), different combinations of interleukins (IL),^87,88^ vascular endothelial growth factor (VEGF),^89^ platelet-derived growth factor (PDGF)^90^ and epidermal growth factor (EGF).^91^ The detection of such growth factors within the niche in vivo has helped to direct development of specific cytokine cocktails. Ligand presentation form may be critical for the outcome, as illustrated by SCF, proven to be more effective in promoting HSCs expansion when bound to the membrane.^92^

Cultivation of NSCs has been performed with the established mitogen basic fibroblast growth factor (bFGF).^93^ Addition of the Tie-2 receptor ligand, Angiopoietin (Ang2), to the culture medium in the presence of Dll4 supported self-renewal and precursor expansion.^94^ Furthermore, cholera toxin,^95^ K+^96^ and high doses of insulin growth factor-1 (IGF-1)^97^ supplementation in the media also enhanced NSCs expansion in vitro.

In addition to the Wnt, Notch and BMP pathways, activation of the EGFR pathway by EGF supplementation is routine in SI and colon organoid cultures.^44,45^ Optimal combinations of factors for stem cell culture conditions of murine and human intestinal samples have been previously detailed, including gastrin, nicotinamide and TGβ1 and p38 inhibitor inhibitors.^45^ In vivo, IGF1 and IL-22 have been found to confer proliferative stimuli to ISCs in response to injury.^98,99^ More recently, clonal ISCs from human foetal colon were grown in two dimensions, in the presence of mouse feeder layers in a similar media to that of 3D organoids, offering an alternative for the study and expansion of ISCs in vitro where the requirement is for homogeneous ISC populations and absence of differentiated cells.^100^ Moreover, adult colorectal samples were successfully expanded as conditionally reprogrammed cells in the presence of rock inhibitors and 2% oxygen in an approach more adaptable to high-throughput studies.^101^

Various soluble factors such as hepatocyte growth factor (HGF), IGF-1, bFGF, PDGF-BB, VEGF and nitric oxide have enhanced MuSCs proliferation.^102,103^ Inhibition p38 associated with enhanced muscle repair in vivo has also promoted MuSCs self-renewal ability in vitro.^104^

Although finding the optimal combination of soluble factors for ASC expansion can be challenging, progress has been made by high-throughput screenings and by the application of cues from pathways activated during development, when stem cell activity is at its highest.

### Neighbouring cells

The cellular components of the ASC may vary according to tissue type, age, steady state or response to injury. Many of the above-mentioned soluble factors are present in the native niches by secretion or membrane-attachment onto ASCs neighbouring cells. These may be other stem cell copies, more committed progenitors, fibroblasts, immune cells, endothelial cells and nerves. Cells associated with ASC niches are therefore likely to produce a host of interactions which regulate ASC function. Some of the essential cell-cell interactions for ASC propagation in specific niches are overviewed below.

Osteoblasts, osteoclasts and fibroblasts comprise the endosteum, considered the osteoblastic niche in the bone marrow. The vascular niche resides within the perivascular space, where endothelial and reticular cells from sinusoids in close proximity to HSCs are considered important players in HSCs homeostasis. Osteoblasts have been implicated as playing a central role in the maintenance of HSCs which reside alongside them, via N-cadherin-mediated cell-cell interactions.^24,105^ These interactions influence HSCs numbers via BMP signalling^24^ and HSCs quiescence via secretion of Ang, TPO, SCF-1 and the chemokine CXCL12.^106^ Endothelial cells (ECs) have been shown to be essential for bone-marrow reconstitution after damage in a VEGFR2-mediated manner.^107^ In vitro, the secretion of Notch ligands and direct contact with ECs enhanced LT-HSCs expansion without exhausting the compartment,^57^ an effect also observed in the presence of Ang2 and Ang3.^108^ Sympathetic nerves and macrophages were also shown to influence HSCs maintenance,^109,110^ illustrating the array of complex interactions involved in niche homeostasis. Cell-ASC heterotypic interactions critical for HSC expansion remains to be explored as tools for proliferation improvement. Nevertheless, it is likely this will become increasingly important for mimicking such complex niche within ex vivo settings.

Both SGZ and SVZ NSCs are located in close proximity to ECs, a region considered as the neurogenic vascular niche in the central nervous system. Endothelial smooth muscle cells, microglia, mature astrocytes and NPCs are all found neighbouring NSCs.^111^ As opposed to the SGZ, SVZ harbours noggin-expressing ciliated cells,^112^ namely, ependymal cells (type-E), which promote cerebrospinal fluid flow, thus allowing NSCs to access multiple neurogenesis modulating factors such as BMPs, Wnt, noggin and retinoic acid (RA).^111^ Further work is needed to understand the difference between NSCs derived from these two different sources and the role of neighbouring cells in promoting their stemness. In fact, co-culturing studies have shed some light on the mechanisms involved in such interactions. Co-culture of ECs with NSCs promoted self-renewal and neurogenesis,^113^ possibly through secretion of factors such as VEGF, BMP, Ephrin-B2 and brain-derived neurotrophic factor,^111^ as could be expected considering the vascular nature of neurogenic areas. Furthermore, a co-culture model investigation aiming to study olfactory bulb putative NSCs has shown that glial astrocytes from the SVZ promoted NSCs expansion via Wnt7a^114^ indicating once again that heterotypic cell interactions might constitute a successful strategy for ASC long-term culture.

Paneth cells are in direct contact with SI ISCs. Working as their niche in intestinal cultured organoids,^15^ they secrete essential factors for ISCs self-renewal, proliferation and homeostasis, such as EGF, TGF-α, Dll1, Dll4 and Wnt3a.^115^ The crucial Wnt3a signal is produced exclusively by Paneth cells and transferred in a membrane-bound, juxtacrine manner.^43^ More recently, in parallel to Paneth cells in the SI, DCCs were demonstrated to function as an ISC stem cell niche in colon organoids, producing mainly Notch ligands.^115^ Some of these factors are also secreted by smooth muscle actin-expressing stromal cells from the intestinal lamina propria^116^ and by other cell types present within ISCs niches. These comprise fibroblasts, ECs, myofibroblasts, mesenchymal cells and immune cells.^116^ In addition to the established multilineage organoid system, two culture systems in 2D using feeder layers have also been described for ISCs expansion,^100,101^ supporting the in vitro use of stromal feeders as a form of emulating niche-driven expansion of ASCs. Supporting cells associated with specific ASCs are therefore likely to produce a host of interactions regulating quiescence, self-renewal and cell cycle entry for multiplying the latter. With further studies, more critical interactions for the proliferation of the ASC pool should be uncovered.

SaCs are scattered as single cells associated with post-mitotic multinucleated muscle fibres under the basal lamina, which constitute their niche.^17^ Myofibres in the SaCs vicinity influence SaCs behaviour via secretion or presentation of membrane-bound factors, such as stromal-derived cell factor 1, a migratory stimulus.^117^ During repair, SaCs are activated and expanded in response to macrophage-secreted factors, as observed in co-culture experiments.^118^ It is noteworthy that ECs are found in every vascularized tissue and that the EC-ASC interaction in multiple niches all point to a critical role of ECs in regulating self-renewal potential. In the muscle, the role of ECs is indeed deemed essential for SaCs maintenance. For instance, ECs from capillaries found in close vicinity to SaCs have been shown to stimulate SaC-derived progenitors when co-cultured.^102^ Although the role of many other cell types has been highlighted in terms of modulating ASC behaviour, it is likely that additional critical interactions will be revealed with further studies addressing more complex multicellular configurations.

### Extracellular matrix composition and biophysical aspects

Structural components of the ECM play a critical part in the ASC niche in vivo. The roles that different ECM components play in the self-renewal, quiescence, proliferation and differentiation have been studied in specific niches and may thus be used to control or direct ASC fate.^119–122^ The ECM that embeds ASCs is composed of structural protein fibres, like collagens and elastin, adhesive proteins, such as fibronectin and laminins, as well as of proteoglycans and polysaccharides. Its shape and composition varies with tissue type, development stage and physiological or pathological processes,^123^ exerting influence on stem-cell fate.^117^

The notion that the ECM is a reservoir of growth factors and morphogens has been vastly reported and evidenced. Many of these proteins will bind to the heparin and heparan sulphate (HS) of proteoglycans and get released as soluble signals during cell-mediated ECM degradation, while others utilize proteoglycans as co-factors to bind their ligand making them readily available to cells.^124^ Aside from growth factor homing and presentation, the ECM has biophysical properties known to play a key role in ASC maintenance. Understanding the interaction between ASCs and their ECM in different niches may enable more successful expansion of these cells in vitro.

#### Integrin-mediated interactions with ASC niche and ECM components

Cells naturally align to topographical features of their surrounding tissue via contact guidance, using cellular projections termed filopodia.^125^ Cell contact and adhesion to the ECM is mediated by the integrin family of transmembrane receptors, which are activated upon binding their ligands, like the peptide motif RGD, commonly found in domains of ECM proteins including collagen, laminin and fibronectin. Once integrins bind to their ligands, they trigger intracellular and extracellular responses. These include, but are not limited to, changes in cell shape via cytoskeleton contraction, integrin clustering on the cell surface forming large focal adhesions and ECM remodelling. The capacity for integrin-mediated dictation of cell behaviour is strongly dependent on the density of ligands^126^ and the number of integrins participating per focal adhesion.^127^ For example, high ligand density (<70 nm apart) causes large focal adhesions with at least four integrins, which results in cells adopting a stretched conformation, whereas low ligand density (>70 nm apart) leaves cells with small focal adhesions and a round conformation.^125^ This indicates that in terms of integrin-dependent association with their ECM, ASCs could be more likely to maintain their stemness when they have small focal adhesions as a result of a low density in fibrillar proteins.

Further supporting this concept is the observation of fibronectin, a critical component of cell-ECM adhesion, found as a compact non-functional soluble form in circulation or assembled into extended fibrils after binding its mechanosensitive RGD motif to cell-surface integrins. As fibrillar fibronectin is one of the first ECM proteins actively assembled upon injury, either by recruiting circulating fibronectin or producing their own,^124,128^ it could be argued that ASC pool expansion to replenish tissue following injury may correlate to fibrillar fibronectin levels.

In the bone-marrow niche, HSC binding to fibronectin via α4- or α5-integrins was essential for replenishing the HSC pool following transplantation^129^ and prolonged ex vivo cultures of regenerating primate HSCs were achieved in the presence of fibronectin.^130^ Whether supplemented on its own^131^ or in combination with laminin,^132^ fibronectin has generally been found to promote clonogenic growth of HSC and HSC-derived progenitors. It has also been reported that tenascin-C, expressed by stromal and endothelial cells in the bone-marrow niche, plays a crucial role in haematopoietic regeneration after myeloablation, promoting HSC proliferation. Accordingly, in vitro cultures of HSCs on a tenascin-C substrate facilitated α9-integrin-dependent stem cell expansion.^133^ Furthermore, collagen type I, the main component of bone which also constitutes the HSC osteoblastic niche, favours a higher amplification of CD34+ HSCs from cord blood.^134^

In the neural SGZ and SVZ of the brain, ECM structures termed fractones make up the NSC niche.^116^ Laminin, one of the three major components of basal lamina, has been reported to improve NPC expansion mediated by α-integrin when compared to fibronectin and the commercially available ECM mix Matrigel^©^.^135^ Chondroitin-sulphate (CS) GAGs were also implicated in NSC proliferation both in vitro and in vivo.^136,137^ Indeed, CS-GAG hydrogels conditioned with FGF have been shown to prolong neurosphere cultures from postnatal rats in vitro.^138^ Similarly, endothelium-derived tenascin-C was considered a critical ECM component for proper neurogenesis,^139,140^ thus possibly facilitating NSC expansion in vitro.

A role for α- and β-integrin-mediated interactions in intestinal culture stemness is suggested by studies which have investigated specific ECM components and ISCs.^141^ Intestinal primary cultures have been extended when placed in collagen I.^121,142^ Furthermore, addition of different types of laminin to such collagen I coatings improved growth of Lgr5+ ISCs and progenitors in 2D cultures for later 3D modelling.^143^ In order to investigate specific ECM components involved in intestinal organoids maintenance, which are normally grown in chemically undefined Matrigel, it was shown that individual addition of fibronectin, laminin 111, collagen IV, hyaluronic acid or perlecan to PEG hydrogels improved ISC proliferation.^46^ Furthermore, binding to HS was reported to activate regeneration via the Wnt canonical pathway in ISCs.^144^

The myofibres where SaCs lie within are rich in collagen IV, laminins and proteoglycans.^145^ Specifically in the muscle niche, fibronectin has been identified as a preferred adhesion substrate for MuSCs, playing a key role in their regenerative potential^146^ while β1-integrin has been considered essential to SaCs homeostasis and self-renewal.^147^ Collagen IV was also found to be necessary for SaCs in response to injury.^148^ Laminin is found not only in myofibrils but also in the juxtavascular niche for SaCs, where it was shown to play a role in the EC-mediated proliferation of SaCs.^102^ More specifically, laminin 521 offered superior results for long-term expansion.^149^ Heparan-sulphate proteoglycans form a hydrogel matrix conferring compressive resistance to the muscle while being freely diffusible for soluble signals. Such properties may be directly related to activation and proliferation of SaCs during muscle regeneration mediated by HS-proteoglycans.^150^

#### Geometry and mechanotransduction

The ECM protein collagen defines the shape and geometry of a given tissue.^151^ As variations of collagen-coated substrates are now more regularly used to expand stem cells,^152^ tracing the collagen signature of the native ASC niche and reproducing that geometry in vitro may offer a significant improvement in ASC expansion. To mention but one example, an individual cell-based computational model of intestinal organoids, which accounted for the bending modulus on the organoid surface, was used to simulate intestinal organoid development in silico.^153^ The work by Buske et al. revealed that the proliferating stem cells on a flexible basal membrane were able to alter the curvature of the membrane. This altered curvature, forming crypt-like domains mimicking the in vivo conformation of intestinal tissue, resulted in changes to the ISC/Paneth cell ratio.^153^ The ability to alter cell specification by mimicking the tissue geometry of the intestinal niche provides evidence for encouraging the development of microenvironmental geometrical cues for ASC proliferation.

Indeed, geometrical features such as pore size in 3D matrices have been explored in ASC culture optimization studies. A study in which HSCs were grown on a porous fibronectin-coated scaffold with microcavities varying between 15 and 80 mm in diameter demonstrated that fitting only individual cells in such cavities promoted HSC multipotency, as evidenced by increased HSC marker expression in response to spatial restriction. However, this effect was limited to culture conditions where HSCs became exposed to low cytokine levels, which is a more faithful reflection of the in vivo bone-marrow niche.^129^ From this model, it could be hypothesized that pore size plays a role in maintaining stem cell potential despite not facilitating expansion, since proliferation rates on porous scaffolds was decreased.

Emerging technologies in the field of tissue engineering and 3D culture can often provide tools by which to develop relevant 3D microenvironments in which to culture cells. Nanofibre technology exploits the sensitivity of cells to the nanotopography of their surrounding matrix, and in particular has been shown to promote self-renewal or differentiation of stem cells in vitro.^154^ Most of the detailed studies focus on ESCs as opposed to ASC populations. It is worth overviewing these trends, as they are likely to be utilized for ASC expansion and culture. The size of nanofibres generated by electrospinning can be controlled and can range size between 1 and 1000 nm in length and as small as 5 nm in diameter. As such, hybrid meshes of natural (e.g. collagen, gelatin) and synthetic (e.g. polycaprolactone) materials can be generated to mimic topographic patterns and alignment profiles of different native ECM matrices. For instance, polycaprolactone/collagen and polycaprolactone/gelatin nanofibre scaffolds were found to promote human ESC expansion in an undifferentiated state.^155^ Both composition and fibre diameter can significantly alter proliferation and differentiation of stem cells. For example, laminin-coated electrospun polyethersulfone (PES) fibre mesh of 283 nm diameter increased NSC differentiation by 40% while 749 nm diameter increased neuronal differentiation by only 20% compared to tissue culture plastic.^156,157^ Functionalized aminated nanofibre PES was also able to largely improve cord blood HSC numbers and engraftment in mice when used as scaffolds for improving such challenging cultures.^158^ These approaches configure powerful tools to unravel the best-fit nanotopographies for expansion of tissue-specific ASCs.

Mechanotransduction is the method by which mechanical stimuli are sensed by cells after which cells respond biochemically and mechanically. The Hippo pathway is a major signalling pathway involved in the control of organ size in animals through the regulation of cell proliferation and apoptosis by nuclear transduction of mechanical and cytoskeletal signals, mediating cellular responses to mechanical forces.^159–161^ The known downstream transducers of the Hippo pathway, transcription co-factors yes-associated protein (YAP) and transcriptional coactivator with PDZ-binding motif (TAZ), behave as sensors and mediators of mechanical cues by altering their localization within a cell.^161^ When the adhesion surface is small between the cell and its soft extracellular matrix, the YAP/TAZ complex is localized in the cytoplasm of cells causing either apoptosis via proteasome degradation or growth arrest. Mechanical loading and extracellular matrix stiffness cause the translocation of the YAP/TAZ into the nucleus of cells leading to active transcription of genes responsible for cell proliferation and differentiation.^162^ Recently, it has been shown that matrix stiffness controls ISC expansion by influence on YAP activity.^46^ If such direct modulation of ASC behaviour is also present in other niches remains to be investigated, especially when considering differences between stiffness and promotion of proliferation in other stem cell compartments, as discussed in the next section.

Although gain of function in YAP has been shown to promote stem cell expansion in intestine and brain tissue of invertebrate models,^159,163^ such observations were not reproduced in the haematopoietic system of mice.^164^ In mice intestinal epithelium, YAP/TAZ seems to induce proliferation in the stem cell compartment, while inducing differentiation in other cell types via different transcription factors.^165^ In muscle, YAP activity is increased in response to injury, resulting in better muscle mass recovery,^166^ possibly via crosstalk with the Wnt pathway in non-quiescent activated SaCs.^167^ Furthermore, regulation of stemness by mechanotransduction has been described in non-adult muscle, where YAP activation by muscle contractility promoted the transcription of Notch, maintaining the progenitors pool number.^168^ Nevertheless, manipulation of this pathway for enhancement of cell propagation remains to be further tested in cultures.

#### Stiffness and elasticity

Stiffness and elasticity of ECM are universal mechanical cues that cells are capable of sensing and responding to. Changes in ECM elasticity can dictate cell behaviour in terms of proliferation or apoptosis, quiescence or differentiation down to a specific lineage.^160^ Perhaps, one of the most striking studies has shown how naive MSCs can be directed down different lineages solely in response to matrix elasticity gradients. MSCs cultured on collagen-coated matrices with a stiffness mimicking native brain (*E_brain_* ~ 0.1 – 1 kPa) exhibit neuron-like features. At increased matrix stiffness, mimicking that of striated muscle (*E_muscle_* ~ 8–17 kPa), cells exert muscle-cell characteristics, whereas at even higher levels of stiffness mimicking bone (*E_bone_* ~ 25–40 kPa), MSCs obtained osteoblast-like morphology.^169^ This study reveals how cells sense matrix stiffness by first being able to pull on the matrix, and second, by transducing the force required to modify their matrix into a signal for morphological change using cytoskeletal motors non-muscle myosin II linked to focal adhesions complexes.^169^

It has been proposed that nuclear entry of transcription factors linked to matrix remodelling is generally regulated by stiffness.^170^ One hypothesis described to explain these observations is that, with increasing collagen content leading to increased ECM stiffness, residing stem cells form a stable actomyosin cytoskeleton, stable focal adhesions and a tension-stable nucleus.^166^ This could prime cells to TGF-β-mediated activation of ubiquitous transcription factors, such as Smads, to translocate to the nucleus and promote matrix remodelling via collagen synthesis and contractility proteins like α-smooth muscle actin, promoting cells down a differentiation fate.^166^ However, ASCs residing within soft ECM would not contract nor stabilize their nucleus tension, possibly remaining undifferentiated.^166^

Furthermore, RA has been reported as a regulator of shuttle transcription factor linked to HSC self-renewal and differentiation.^171^ Of all the gene targets regulated by RA, lamin-A has attracted the most attention as variations in its expression have a key role in ASCs mechano-responsiveness.^172–174^ Lamin-A is an intermediate-filament protein that controls nuclear deformability, maintains DNA stability and couples mechanical stimulation to biochemical signalling in stem cells. Its level is proportional to primary tissue matrix stiffness (0.1–40 kPa) and increases in cultured stem cells with increasing matrix/hydrogel stiffness.^170,173^ Importantly, A-type lamins have also been implicated as intrinsic modulators of ageing in multiple ASC and their niches via the Wnt canonical pathway, the TGF-β pathway and retinoblastoma transcription factor pathway, the latter more extensively studied in SaCs.^175^ Further studies on mechanical regulation of stemness will allow a deeper understanding on the molecular mechanisms of geometry and stiffness translation to cells. Nevertheless, the relationship between stiffness and ASC propagation has been previously explored in the four niches presently discussed.

Although extracted bone marrow is generally considered a soft tissue (<0.3 kPa),^176^ bone is rigid (>1000 kPa), whereas endosteal-like ECM secreted by cultured osteoblasts is stiff (20–40 kPa).^177^ Therefore, this varied range of stiffness found in bone marrow,^178^ as well as the existence of different niches (e.g. vascular and osteoblastic) and ASC populations (e.g. LT-HSCs and ST-HSCs), could explain non-concordant results in the literature, where both 0.8 kPa^179^ and 12.2 or 30.4 kPa^180^ have been reported as the optimal modulus for ex vivo culturing of HSCs.

Variations in stiffness play an important role even in one of the softest tissues in the body, the brain, whose stiffness changes with age and varies from one region to the other. The juvenile or developing brain (~0.04 kPa) is not as stiff as the adult brain (~1.2 kPa). Studies have reported a proliferation peak of NSCs when grown on substrates of 1–4 kPa stiffness, whereas differentiation down the different neural progeny has been observed on both softer (<1 kPa) or stiffer substrates (>7 kPa).^172,181^

Using intestinal organoids, Gjorevski et al.^46^ have elucidated that culture within a stiff matrix (1.3 kPa) promotes enhanced proliferation and survival of ISCs, whereas culture within a soft matrix (300 Pa) results in poor proliferation and enhanced differentiation capability, conversely to the hypothesis that lower stiffness would promote stemness.^166,170^ This has resulted in the development of dynamic 3D matrices for organoid culture in which stiffness changes over time to ideally encourage expansion of cells in the initial stage, followed by differentiation.^46^

Studies focusing on the MuSC niche have demonstrated that MuSCs retain their capacity for self-renewal in vitro when cultured on 12 kPa hydrogel substrates as opposed to culture in plastic (~10^6^ kPa).^18^ Despite further evidence showing that primary myoblasts present higher proliferation in stiffer gels (12 and 45 kPa) when compared to softer gels (1 kPa),^182^ other studies indicate that 2 kPa would be ideal for expanding the proliferative pool of muscle progenitors.^183,184^ The fact that aged and damaged myofibres are stiff (18 kPa) and that gels presenting such values were inhibitory for proliferative MuSCs should be taken into account during MuSCs growth protocol optimization.^183^ Increasing stiffness beyond optimal expansion modulus could mimic fibrotic conditions,^185^ generally considered counterproductive in terms of cell propagation.^186^ Stiffness related to collagen VI content has also been demonstrated to play a crucial role in SaCs self-renewal, since muscle tissue depleted of collagen VI had significantly reduced stiffness, resulting in impaired tissue regeneration. Nevertheless, this effect was rescued by reinstating the expression of collagen VI.^148^

There is an emerging body of research focusing on the relationship between not only matrix stiffness and but also elasticity on ASC fate. Controlling matrix elasticity in vitro has been attempted using microfabrication and microfluidic technology to assess cellular responses to matrix stiffness gradients.^187^ For example, a microfluidic-based lithography channel was developed containing a hydrogel substrate expressing a stiffness gradient that was generated by introducing polyethylene-fibrinogen and different amounts of polethylene glycol diacrylate (PEGDA) into the channel.^188^ Also, a micro-fabricated post-array made of polymer polydimethylsiloxane (PDMS) and controlled by a magnetic field was used to exert precise elastic forces onto cells in order to investigate how cells would sense these forces.^189^ This advocates for the mechano-sensitivity of cells and emphasizes that careful selection of in vitro culturing conditions is necessary when growing ASCs, since they exert mechanical forces to their attached ECM and cell-fate decisions are based on the feedback gauge.

#### Oxygen

There is general consensus that a low oxygen state maintains stem cell quiescence and self-renewal capability. This is based on the observation that mammalian cells in adult tissues undergo aerobic metabolism and thus generate reactive oxygen species resulting in oxidative stress. This in turn leads to DNA damage and mutations, which accumulate over time.^190^ To maintain stem cells in an undifferentiated and unmutated state, it is hypothesized that the controlled environment of tissue-specific niches protects cells from oxidative stress. There is emerging evidence to suggest that many types of stem cells rely on anaerobic glycolysis to maintain quiescence while limiting DNA damage.^191^ Stem cells reside in a protective niche where oxygen levels fall below those of surrounding tissue, offering a distinct anatomical compartment in which cells can maintain low rates of proliferation and limit oxidative stress.^190^ ASCs within hypoxic environments up-regulate hypoxia-inducible factor (HIF)-Iα which in turn promotes glycolysis.^191^ Low oxygen and transcription factors expressed in response to hypoxia have been found to be involved in maintenance of stemness.^191–193^ Additionally, HIF-2α has also been implicated in the regulation of stem cell master genes SOX2, NANOG and OCT4.^194^ All of this has implications for the way in which stem cells are cultured. Maintaining the biomimetic niche characteristics as we study cell populations is paramount to gain meaningful insights into cell behaviour.

The oxygen range accepted to represent physiological hypoxia in tissues within the body is between 2% and 9%, depending on tissue type.^195^ Compounded by the fact that direct measurement of oxygen tension in tissues is difficult, very often, levels of hypoxia are extrapolated by indirect data which mainly looks at the upregulation of HIF-1α.^196^ Nevertheless, this may not be a direct reflection of hypoxia, as HIF-Iα may be regulated by other factors.^197^

Recent direct measurement of the oxygen concentration within the bone-marrow cavity of mice, the niche for HSCs, has revealed that levels of O_2_ lie between 1.3% and 4.2%.^196^ Although this may seem surprising given the dense vascularity of the bone marrow, this is countered by the high cellularity and thus high consumption of oxygen by proliferating haematopoietic cells.^196^ It has thus been suggested that cellular consumption of oxygen dictates the hypoxic niche. Culturing LT-HSCs at 1% oxygen promotes a decrease in the proliferation rate and an accumulation in G0, but increases their engraftment potential.^192^ This suggests that the low microenvironmental oxygen tension maintains these cells within a protective niche while retaining their self-renewal potential.^192^ This study adds to the significant body of work on the influence of oxygen tension on stem cell quiescence, where low oxygen tension is considered a niche characteristic essential for the maintenance of the undifferentiated states of embryonic and other stem cell types.

In NSCs, there is evidence to suggest that culturing cells in hypoxic conditions, mainly between 2.5% and 5% promotes self-renewal.^198^ Interestingly, the oxygen levels in brain vary from 14% to 0.55%.^199–201^ This large range means cells in different brain compartments are exposed to varying oxygen levels. Moreno and colleagues have elucidated some optimal oxygen ranges to culture NSCs, shown to self-renew and proliferate at 5% oxygen. In this study, the authors showed that the calcium regulated transcription factor *Nfatc4* signalling axis acts as a major regulator of NSC self-renewal and proliferation both in vitro and in vivo.^202^ Levels of hypoxia as low as 1% oxygen tend to lead to apoptosis of NSCs.^198^

Accordingly, ischaemic preconditioning aiming to protect SI against reperfusion injury has driven upregulation of Lgr5, increasing formation of crypt organoids in vitro while upregulating HIF1α and Wnt/β-catenin.^203^ Although oxygen levels were not measured in this study, conditionally reprogrammed cells from colon tissue seem to better expand in a 2% controlled oxygen incubator.^101^

Looking at muscle ASCs, evidence suggests that hypoxic levels as low as 1% oxygen maintain stemness in murine SaC-derived primary myoblasts.^204^ This study found that culture of primary SaCs at 1% oxygen resulted in the upregulation of Pax7, a key regulator of SaCs self-renewal. Other studies using various MuSCs populations are in agreement with this. In murine muscle myoblasts, 1% hypoxic conditions inhibited the differentiation of these cells, but this process was found to be reversible.^205^ Similarly, culture of bovine SaCs at 1% oxygen enhanced proliferation rates.^206^ Hypoxic conditioning at 5% oxygen was also reported to stimulate proliferation of human skeletal precursor cells.^207^

The importance of the hypoxic element on the ASC niches is thus evident in its direct influence on retention of stemness by cells and prevention of differentiation.

## Advances in microenvironment biomimicry for ASC proliferation

**Figure 1.**
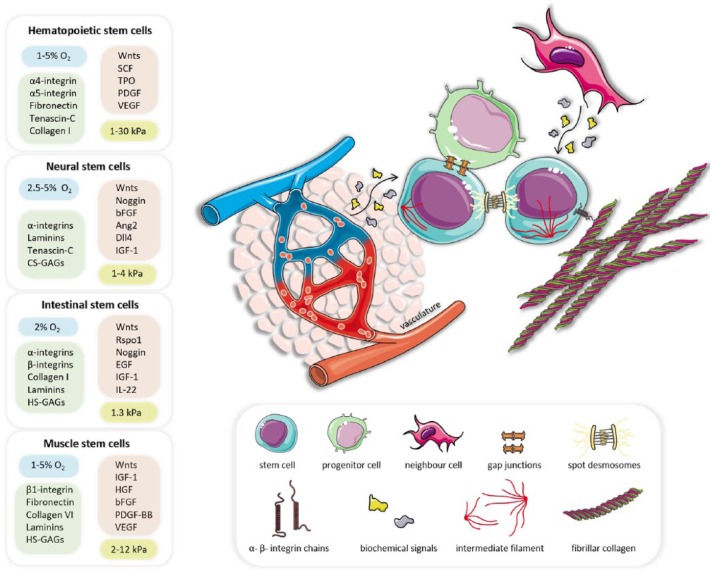
Schematic representation of generic adult stem cells (ASCs) niche features, including ASCs, their progenitor cells, neighbour cells, the vasculature and the extracellular matrix. Adult stem cells sense and respond to their surrounding extracellular matrix via integrin-mediated adhesion, depicted as α- and β-integrin chains, together with cytoskeletal intermediate filaments and intercellular spot desmosomes. Paracrine signals and growth factors are secreted by the vasculature and proximal progenitor or neighbouring cells, overall influencing ASC fate. The overarching biochemical, biophysical and biomechanical features discussed and referenced in this review have been distilled down to a list of conditions reported to promote ASC expansion in the four tissue-specific niches of interest. Elements within the boxes comprise general recommendations for oxygen levels (blue), ECM components (green), biochemical factors (orange) and stiffness module (yellow) to be used in specific ASC cultures. This figure was prepared using elements from Servier Medical Art.

There has been a concerted effort towards recreating biomimetic features in vitro to direct ASC culture. Soluble factors play a critical role in determining ASC self-renewal, proliferation and differentiation, but they play a role alongside and in conjunction with other microenvironmental features. Much recent work has focused on manipulating physical microenvironmental features to modulate ASC fate and these physical components also regulate the release of soluble factors. The main findings highlighted in this review for bone marrow, neural, intestine and muscle tissues ASC niches are overviewed in Figure 1. In addition to biochemical and biophysical factors, other promising advances are likely to impact the field, such as the introduction of more physiological complexity to models by the use of microfluidics, innervation/electric stimuli, high-throughput culture testing and the use of dynamic and nanoscaled matrices.

2D culture of bone-marrow stromal cells is a well-established technique to support in vitro short-term expansion of non-adherent bone-marrow cells.^208^ Aiming to improve the necessary expansion of HSCs for clinical applications, engineering strategies have been tested with additional components such as scaffolds, cytokines and supporting stromal cells. PEG-based hydrogels with immobilized SCF and IFN-y enhance the expansion of mouse HSCs and progenitors; however, transplantation assays to confirm the reconstitution ability of expanded HSCs remain to be performed.^209^ A newly developed scaffold-free automated system with feedback control was developed after in silico prediction of negative regulators of HSC compartment expansion. A tuneable mode of media regulation and a fed-batch feeding strategy that keeps cells at low densities instead of perfusion, promoted an increase in HSCs from cord blood in 11 times after 12 days of culture.^210^ In another study, PDMS scaffolds filled with collagen I were implanted, and thus conditioned, in mice for 8 weeks and explanted for use as engineered bone marrow in a microfluidic device. These were seeded with HSCs and resulted in functional and proportional expanded HSCs populations within 1 week. This model was successfully tested for toxicology assays and for studying the bone-marrow niche while controlling cell populations.^211^

Researchers are now able to engineer arrays of micro-wells with robotic protein spotting and topographical micro-patterning of hydrogels, which clearly demonstrate that laminin and fibronectin in specific defined amounts improve mouse NSCs expansion.^212^ Further methods to expand NSCs include 3D culturing in PDMS-based microfluidic devices.^213^ These devices hold a central channel, seeded with NSCs and collagen type I hydrogels, which are then surrounded by adjacent channels where cultured brain vascular cells form tube-like structures. This set-up highlights the importance of the presence of vascular cells, or other important cell-cell interactions, resulting in increased NSC survival, neurosphere formation and size.^213^

Long-term culture of ISCs has been extensively demonstrated by the Matrigel-based organoid systems, which allow the in vitro production of both stem and differentiated intestinal lineages.^44,45^ The use of synthetic chemically defined 3D matrices generating the same effects as Matrigel could overcome problems imposed by this product’s use, such as its animal origin, unknown precise composition and unsuitability for clinical applications. Recently, synthetic ECM analogues produced by RGD-functionalized PEG hydrogels with different stiffness were found to be appropriate platforms for intestinal organoid cultures. Moreover, ISCs expansion was optimal under higher stiffness while matrix softening could promote differentiation, highlighting the utility of using dynamic matrix strategies for a tight control of ASC fate.^46^ Such strategies can now be applied to other established organoid cultures in order to substitute Matrigel and define which conditions might increase ASCs number before inducing their commitment to more differentiated lineages.

Aiming to develop a culture model more reflective of the native environment, Serena and colleagues have coupled electrical stimulation to SaCs cultures on 3D porous collagens scaffolds.^214^ Using this method, both cell proliferation and myogenic potential were improved via nitric oxide release promoted by electrical stimulation before implantation in mice.^214^

The aforementioned examples illustrate that, despite the accumulated knowledge on ASC niche composition and their role in laboratory-based expansion methods, much remains to be explored in order to overcome the notorious difficulty to achieving long-term propagation of ASCs.

## Concluding remarks

Expanding ASCs from tissue samples for longer periods while maintaining their stemness in order to obtain therapeutic and high-throughput testing cell numbers is a much needed tool for stem cell biology and tissue engineering. By reviewing four exemplar ASC niches with different cell turnover rates, we have overviewed how soluble factors, 3D matrix composition, stiffness and oxygen tension all act on ASCs.

In some instances, it is clear to see how the native microenvironment still resonates with cell behaviour, which includes how ASCs react upon stiffness changes and oxygen levels. It is also becoming clearer that representing features of the native microenvironment can be a good starting point to recapitulate in vivo behaviours in vitro. Still, obtaining higher ASCs number from small amounts of starting samples can be challenging, especially for tissues with native low turnover rates and low repair abilities, which may need to be expanded ex vivo to much higher rates than in vivo. Understanding the molecular mechanisms which drive transition between quiescent and proliferative states of ASCs is thus crucial for optimization. Likewise, searching for biological cues from development and injury response may help the development of fitter strategies to bring scientific community closer to effectively use ASCs in basic and applied biomedical research.
